# Exploring Narrative Structure and Hero Enactment in Brand Stories

**DOI:** 10.3389/fpsyg.2018.01645

**Published:** 2018-09-19

**Authors:** José Sanders, Kobie van Krieken

**Affiliations:** Centre for Language Studies, Radboud University, Nijmegen, Netherlands

**Keywords:** archetype, brand story, brand video, catharsis, enactment, heroism, narrative, phronesis

## Abstract

This study examines how audiovisual brand stories both invite and enable consumers to enact heroic archetypes. Integrating research on the archetypal structure of narratives with research on the event structure of narratives, we distinguish singular plot stories (i.e., stories that *show* a Hero’s Journey) from embedded plot stories (i.e., stories that not only show but also *tell* one or more Hero’s Journeys) and develop a conceptual and narratological framework to analyze their structural elements. Application of the framework to 20 brand stories representing 8 different brands reveals meaningful variation in elements between the singular plot stories and embedded plot stories. Differences in the expression of archetypes and event structure are argued to evoke different types of Hero enactment which in turn result in different outcomes. We specifically hypothesize that the enactment of heroic archetypes in singular plot stories primarily results in *catharsis* (pleasure), whereas the enactment of heroic archetypes in embedded plot stories primarily results in an outcome we describe as *phronesis*: a form of moral sense making of the self that advances one’s practical wisdom and prudence. The final section of the paper discusses how cathartic and phronetic outcomes of hero enactment may foster the psychological bonding between brand and consumer, and invite consumers to align their moral values with the values that are reflected by heroic character traits. The central aims of the analysis presented are to provide an exploration of narrative phenomena in a reasonably broad range of brand story videos and foremost to provide a conceptual framework with an applicable instrument suited to analyze relevant categories in these brand stories. The present study is interdisciplinary in its approach to a contemporary, developing marketing phenomenon, applying psychological modeling of archetypes and heroic values with narratological insights on perspective-taking and story structure. Its contribution is to systemize, from a narratological viewpoint, how various narrative archetypes in brand video stories may contribute to the development of brand-consumer relations.

## Introduction

Stories are told in every domain of social life: parents tell their children bedtime stories about dragons and castles, teachers tell their students stories about history and society, musicians sing their fans stories about budding and fading romances, organizations tell their stakeholders stories about their past performances and future goals, and novelists tell their readers stories about possible lives in possible worlds. The human inclination to communicate via stories rather than expository information is often explained by the presumption that narrative is deeply rooted in both our cultural and biological makeup (e.g., Boyd, 2009; Niles, 2010). It has in fact been argued, most notably by Bruner (1986), that narrative is a specific mode of thought, different from logical reasoning, on which we rely in making sense of the world and the self (see also Bruner, 1991, 2004).

**FIGURE 1:**
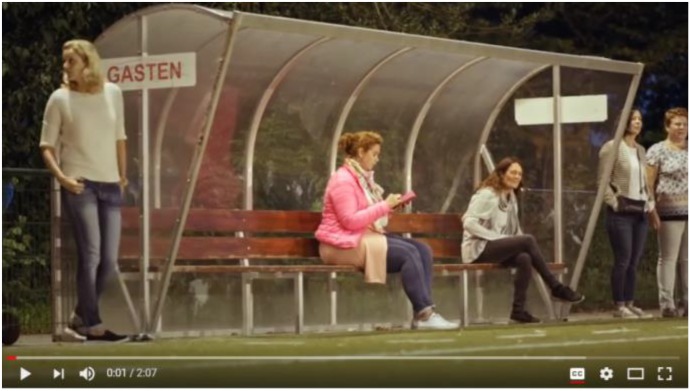
Start of the Pickwick story showing the mother occupied with her smartphone. Image reproduced with the permission of Jacobs Douwe Egberts.^2^

While each story is unique and stories vary greatly in content and style, they share a foundation of recurring prototypical elements called archetypes. Perhaps the most well-known character archetype is that of the Hero who embarks on a quest to find a treasure and faces one or more obstacles along the journey while being supported by a helper. This template of *The Hero’s Journey* (Campbell, 1949) can be recognized in stories as diverse as ancient poems such as The Odyssey, Hollywood movies such as the Indiana Jones series, and brand advertisements. An example of the latter category is a Dutch story from 2016 by tea brand Pickwick.^1^ The audiovisual story is part of a campaign entitled “Tea Topics” which released a series of brand videos in which customized tea labels play a central role. Beau’s Tea Topic is about a young girl who is frustrated that her mother, whom she used to be very close to, has been spending an increasing amount of time with her smartphone and less with her daughter (see **Figure 1**). In the filmed narrative, she invites her mother for a walk in the woods, and sitting down for a rest she hands her mother a tea bag with a question printed on its label: *Could you please turn off your phone for 1 day?* The girl subsequently explains her frustration, upon which the mother makes a promise to change her behavior. After this exchange, they drink tea together and walk back arm in arm. In this story, the archetypal role played by the girl is that of the Hero, whereas the brand fulfills the archetypal role of the Helper that supports the Hero in completing the difficult quest of confronting her mother with her behavior so as to restore their relationship.

Brand stories like the Pickwick story deviate from traditional marketing and advertising efforts in that the promotion of the product or service is of secondary importance (Laurence, 2018). The central goal of brand stories is to “design brand experiences that stretch beyond mere products and price points” (Smith and Wintrob, 2013, p. 37). A specific consumer experience is that of *archetype enactment*, which refers to the experience of adopting the role of an archetypal character and vicariously performing the actions central to the brand story (Woodside et al., 2008). This enactment, which has both a psychological and an embodied component, may result in pleasure and in sense making (Woodside, 2010). Although pleasure and sense making are both consumer-centered (rather than brand-centered) outcomes, they differ in that the former is dominantly emotional in nature and the latter dominantly rational. The present study explores how brand stories aim to evoke these outcomes by scrutinizing the narrative structures of brand stories. Specifically, it will be studied how narrative brand videos both invite and enable viewers to enact heroic archetypes. To that end, research on the archetypal structure of narratives (Campbell, 1949) is integrated with research on the event structure of narratives (Labov and Waletzky, 1967; Labov, 2010), leading up the development of a conceptual framework to analyze audiovisual brand stories. We subsequently analyze 20 brand stories in an initial assessment of the framework’s viability. Results of the analysis give rise to the generation of testable hypotheses about the relation between the archetypal and narrative structure of brand stories, the nature of consumers’ hero enactment, and outcomes of pleasure and sense making. As such, this study aims to further develop the evolving field of Heroism Science (Allison, 2016).

### Narrative Archetypes

Research on narrative archetypes is rooted in the work by Jung (1916) and Campbell (1949). Central to their work is the understanding that part of the human psyche is universal in that it houses “content and modes of behavior that are more or less the same everywhere and in all individuals” (Jung, 1916, p. 4). This “collective unconsciousness” is structured around recurrent patterns and symbols called *archetypes*. Archetypes can be *events*, such birth and initiation, *figures*, such as the hero and the trickster, or *motifs*, such as the apocalypse and the deluge. Jung (1916) proposes that archetypes are both innate and universal, which, as he argues, explains for their capacity to organize human experience and to guide people through the cycle of life, from the early stage of being born and parented to the final stage of preparing for death. Archetypes are in this view understood to be unconscious concepts that change into “conscious formulae” through expression in cultural artifacts such as myths and fairytales (Jung, 1916, p. 4).

The expression of an archetype in cultural artifacts reinforces the archetype through repetition. In narrative in particular, the same archetypes are evoked over and over again, across time as well as across cultures. As argued by Campbell (1949), virtually all narratives are structured around a limited number of archetypes. A highly dominant structure is that of *The Hero’s Journey*, a generic template of archetypal events, figures, and motifs of which the details vary per story. This “monomyth” is described by Campbell (1949, p. 23) as follows:

“A hero ventures forth from the world of common day into a region of supernatural wonder: fabulous forces are there encountered and a decisive victory is won: the hero comes back from this mysterious adventure with the power to bestow boons on his fellow man”.

Instantiations of The Hero’s Journey include several or all of seventeen stages that are divided into three acts: the departure, the initiation, and the return. The *call to adventure* marks the beginning of the journey, when the Hero is “drawn into a relationship with forces that are not rightly understood” (Campbell, 1949, p. 42). Upon responding to the call, an encounter with a *supernatural aid* is likely to follow that provides the Hero with “amulets against the dragon forces he is about to pass” (Campbell, 1949, p. 59). In the Pickwick story, the brand provides the girl with the courage to confront her mother with her egocentric behavior by means of a tea bag which has the message printed on the label.

**FIGURE 2:**
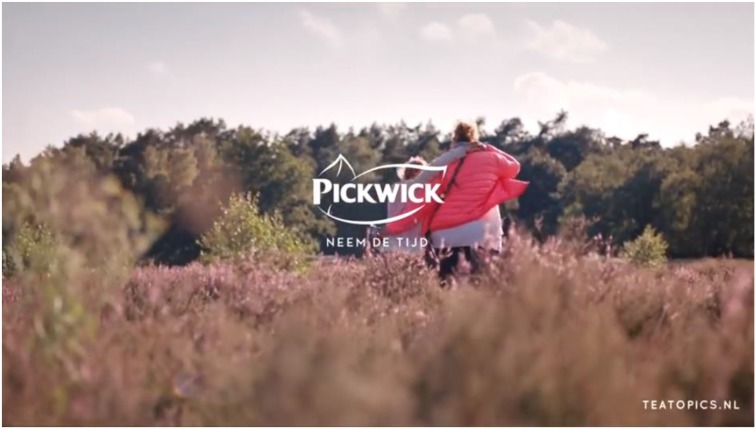
End of the Pickwick story showing mother and daughter walking away arm in arm. Brand slogan *Pickwick - Neem de Tijd [Take the Time]*. Image reproduced with the permission of Jacobs Douwe Egberts.

In the initiation stage the Hero faces and overcomes difficult obstacles, with the result of being rewarded with a treasure. Drinking tea with a mother who is not distracted by her smartphone is Beau’s treasure in the Pickwick story, received after overcoming the obstacle of having a difficult conversation about her feelings. The Hero then returns home, transformed by the journey. Pickwick depicts this return in the final shot of the video when Beau and her mother walk away from the camera, now arm in arm (see **Figure 2**). Notably, both characters have undergone a transformation that can be characterized as emotional in nature (Allison and Smith, 2015): Beau has transformed from frustrated to courageous and happy and the mother has transformed from a distracted and disconnected into an attentive and committed person. The mother has in fact undergone the greatest transformation, which exemplifies how heroes need not always be significantly transformed themselves but can express their heroism by sparking transformations in others (Allison and Goethals, 2017). In addition, viewers may identify either with the narrative’s central hero or with another character depending on their own cultural or societal position, for instance – in the Pickwick story’s case – as a daughter or as a mother.

Both Jung’s work on archetypes and Campbell’s work on the Hero’s Journey have been criticized for providing a gendered, i.e., dominantly masculine, account of heroism (e.g., Goldenberg, 1976; Nicholson, 2011). The concepts developed in their work are nevertheless useful in understanding why people are naturally attracted to stories: their generic template shows similarities with the life cycle of individuals. Like the Hero in a narrative, individuals undergo transitional stages in their lives. These include changes resulting from life experiences (separation, moving elsewhere, job change) as well as life transitions and changes in societal status (adolescence, relationship, parenthood, old age), that are ritualized and celebrated in *rites of passage* (such as wedding, baptism, first Communion, bat and bar mitzvah, funeral; see Van Gennep, 1909). The resemblance of life cycles and their transitional phases makes stories universally resonating and explains why they can generate deeper insight into the self and the other. Research on archetypes provides us with a framework within which to deepen this understanding and in particular with the language and concepts to capture the nature of the relation between story characters and audience members. The framework has proven useful in the analysis of stories as diverse as narrative video games (Buchanan-Oliver and Seo, 2012) and popular songs (Almén, 2003). The present study employs and extends the conceptual framework to examine the characteristics of audiovisual brand stories with the goal of gaining insight into as well as formulating expectations about the experiences these stories elicit in consumers. Understanding the relation between narrative archetypes, archetype enactment, and narrative outcomes can be advanced by examining the archetypal structure in combination with narrative structure, i.e., the presentation of elements that together form the story plot.

### Story Structure

Research on narrative plot structures has a long history and is similar to research on narrative archetypes in its focus on recurring story patterns. Much work in this area has been concerned with folktales. Propp (1928), for example, identified 31 elements that are characteristic of Russian fairytales. This inventory includes an *absentation* that marks the beginning of a fairytale in which one of the characters leaves home, a struggle, a victory, a return, and, finally, a wedding. This classification has, indeed, been of influence to Campbell’s (1949) work on The Hero’s Journey.

Departing from narratives in dialog, a complementary structural classification of story elements was developed by Labov and Waletzky (1967). Their influential categorization is based on the notion of narratives’ tellability, that is, the degree to which the story’s plot allows the narrator to claim interactive space and attention. Analyzing conversational stories, they distinguish six core elements that together form the essential story plot (Labov and Waletzky, 1967; Labov, 2010). The *orientation* refers to the opening of the story world by introducing the setting and characters (who? what? where? when?). The first scenes of the Pickwick story constitute the orientation by introducing the characters and the story’s setting. Images of the Heroine Beau, playing a match with her soccer team, her father and her mother are accompanied by Beau’s voice: “*I am Beau. This is my father and he is my soccer coach. And this is my mom and she is always busy with her phone*.”

**FIGURE 3:**
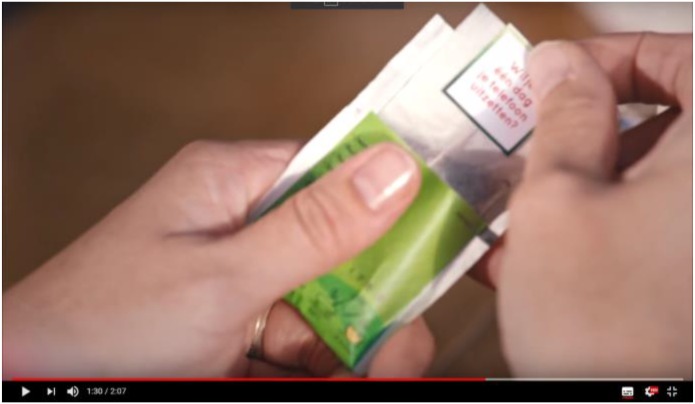
Still from the *Pickwick* story showing the mother opening the packaging and revealing the customized question printed on the tea bag label. Image reproduced with the permission of Jacobs Douwe Egberts.

The *complicating actions* are the events leading up to the *critical event* of the story: the central event that lends the story its ‘tellability,’ i.e., the reason and justification for the story to be told. The function of the complicating actions is to lead the viewer to the critical event: Beau asking her mother, by handing her a customized tea bag label, to turn her phone off for 1 day (see **Figure 3**). Notably, the critical event is the first part of the story in which Pickwick plays a role. At this crucial point, the tea bag is shown from the perspective of the mother as if we are looking through her eyes. We read along with her the question printed on its label: “*Could you please turn off your phone for 1 day?*”

**FIGURE 4:**
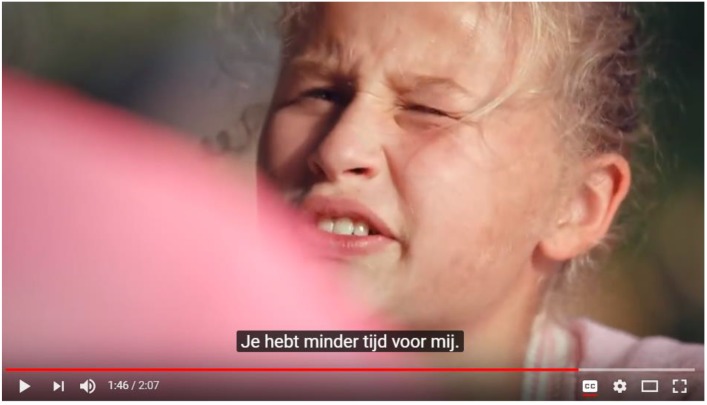
Still from the Pickwick Story showing Beau who, after her mother has read the tea bag label, tells her that she should spend more time with her [literally*: You have less time for me*]. Image reproduced with the permission of Jacobs Douwe Egberts.

The *resolution* refers to the story’s outcome. Beau’s mother responds positively to the question, after which Beau explains that she would like some more attention. After a brief moment of reluctance, her mother agrees once more to change her behavior. During this dialog, the camera switches from over mother’s shoulder when Beau is talking to over Beau’s shoulder when her mother is talking, thus signifying the importance of listening to one another. Then a close-up of two cups of tea is shown, which communicates to the consumer that drinking tea together provides a good opportunity for listening and talking (see **Figure 4**).

The *coda* is the moment where the connection is made with the here-and-now speech situation (in interaction, serving to offer the other interlocutor an opportunity to take over the turn). Thus, it refers to the transition from the story world back into the here-and-now. The final scene of the Pickwick story (see **Figure 2** above) constitutes the coda by showing Beau and her mother walking away from the camera. The brand’s logo is shown along with the slogan “Take the time.” Here, the Pickwick brand functions as a bridge between the story world and the here-and-now world of the viewer in which the brand’s slogan functions as an advice to the viewer: *[You] take the time [in your own situation].*

**FIGURE 5:**
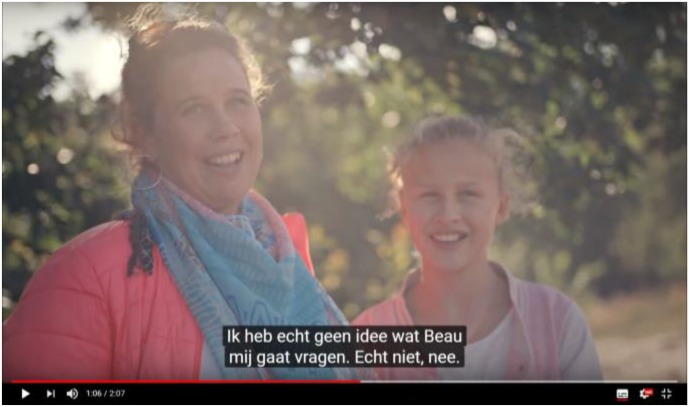
Still from the *Pickwick* story showing Beau and her mother before the critical event, looking into the camera and the mother talking into the camera. [Literally: “*I really have no idea what Beau is about to ask me. Really not, no*.”] Image reproduced with the permission of Jacobs Douwe Egberts.

Throughout a story, *evaluations* can be used that signify the character’s or the narrator’s comments on the events. Evaluations are typically quotations expressing personal experiences (Labov, 2010), such as the emotional impact of the story events or the considerations taken into account when facing a dilemma. The critical event of the Pickwick story is preceded by an evaluation (see **Figure 5**). This evaluative scene shows Beau standing next to her mother, both looking in the direction of the camera, with the mother stating she has no clue what her daughter is up to. Such evaluations, spoken outside the kernel narrative, serve to facilitate the viewers’ identification with particular narrative characters’ experiences – typically extraordinary or impactful experiences that justify the story to be told (cf. Bell, 1995; Labov, 2010). This will be elaborated in the next section.

### Narrating Voices

Audiovisual brand stories such as the Pickwick story of Beau invite the viewer to enact narrative characters’ acts and emotions. This is facilitated not only by visual elements *showing* the story, but also by auditive elements such as speech and textual elements *telling* the story. Visual, auditive and textual elements typically interact in brand videos in representing a story from various – possibly intertwined – perspectives (Sweetser, 2017). For instance, characters can talk to each other within the story or to him- or herself, but alternatively, the character can speak either directly to the camera, implying they talk to an invisible person standing next to the camera, or directly to the viewer in a voice-over or text-over while footage of the past or future experiences of the character is shown.

**FIGURE 6:**
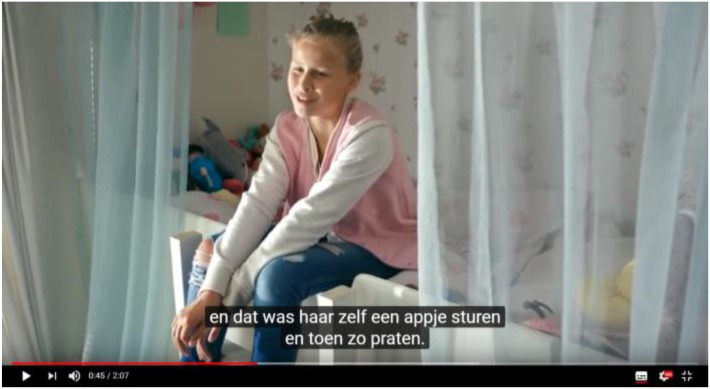
Still showing Beau talking to an invisible interlocutor in her bedroom, telling that the only way to reach her mother was [Literally: “*to send her an app and talk to her that way*”]. Image reproduced with the permission of Jacobs Douwe Egberts.

These distinctive positions steer different, genre-connected expectations. Two main brand story genres can be distinguished: singular narratives and embedded narratives. In a singular (fictional) narrative, the story is *shown* by events that tell themselves (De Jong, 2014, p. 4), played out by characters who navigate on a narrative timeline unconnected to the viewer; direct interaction with the film maker and/or viewer is not part of the narrative world. Such interactions are, by contrast, characteristic of embedded narratives. In an embedded narrative, characters refer to themselves and their opinions in interaction with the film maker and/or the viewer (De Jong, 2014, p. 4). This indicates that the (most often non-fictional) characters are not separated from, but connected to the viewers’ time line (Sanders and Van Krieken, forthcoming). Inserting such film maker- or viewer-directed utterances supports and legitimizes the narrative’s credibility, specifically when *evaluations* are brought in: moments where characters *tell* about their thoughts, feelings and strivings. For instance, the transition from the orientation to the complicating actions is in the Pickwick story marked by a change in setting from the soccer field to Beau’s bedroom (see **Figure 6**), where she explains to an interlocutor who is not visible that recent changes in her mother’s behavior have negatively affected their once close relationship.

This scene bears resemblance to what has been identified as a legitimizing scene in written newspaper narratives (Van Krieken and Sanders, 2016; Van Krieken et al., 2016). In such news stories, which reconstruct past news events (such as a crime or disaster), frequent transitions take place to a setting – typically a press conference, interview, or court session – in which the news actors elaborate and reflect on these events. Such settings are conceptualized as legitimizing because they serve a function of demonstrating that the journalist rightfully and truthfully reconstructs the narrative sequence of news events. Likewise, the scene in Beau’s bedroom fulfills a legitimizing function in that it introduces Beau as a real person with a real problem, thereby lending the story authenticity and credibility.

**FIGURE 7:**
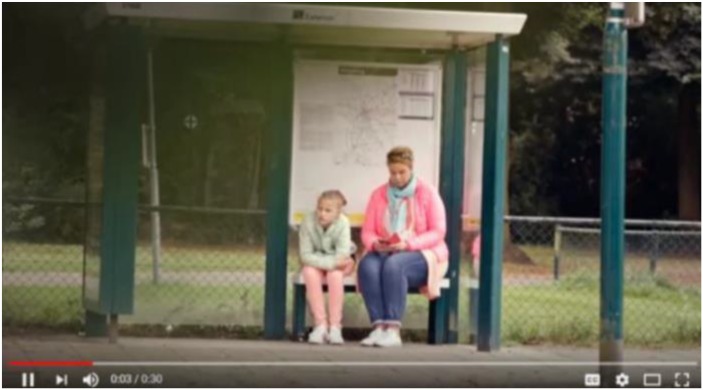
Still from the Pickwick story showing Beau sitting next to her mother at a bus stop. Image reproduced with the permission of Jacobs Douwe Egberts.

The story’s credibility is further enhanced by subsequent short clips that support Beau’s speech and that are ostensibly captured by a hidden camera: a clip showing mom sitting in the living room, watching her phone again and not paying attention to her daughter coming in and giving her a kiss; and a clip showing a clearly frustrated Beau at a bus stop, sitting next to her mother who is watching her phone (see **Figure 7**).

During these scenes from various locations, Beau’s voice continues to narrate about the recent estrangement between her mother and herself, thus supporting the tellability of the story of which the climax is about to be shown. The embedding evaluative scenes invite the viewer to identify with the Hero character’s authentic experiences. Embedding a story plot puts a focus on a particular character as the Speaking Hero whose voice interacts with the viewer in *telling* aspects of the story on top of what is *shown*, thus offering an instrument to further enhance identification with brand stories’ Heroes. In branding and marketing literature, such identification by consumers is defined in terms of Hero enactment.

### Hero Enactment

Branding experts’ interest in narrative archetypes has increased exponentially over the past two decades, as witnessed by a growing number of professional books promoting storytelling as a communication mode capable of creating strong brands (e.g., Martin, 2010; Gioglio and Walter, 2018). Practitioners and scholars alike consider narrative archetypes effective in establishing meaningful psychological relations between brand and consumer (Herskovitz and Crystal, 2010; Nelson, 2018). These relations emanate through the identification of connections between the narrative of the brand and the autobiographical narrative of the consumer (Ochoa and Lorimer, 2017). Narrative archetypes are instruments to disclose as well as interpret these connections via a process of identity mapping. Archetypes communicated by a brand express its identity and resonate therefore most with individuals whose (desired) identity is, at least at that specific moment in their lives, composed of the same archetypes. In other words, consumers identify with brands whose identities map onto their own identities in terms of its archetypal components. This *consumer-brand-identification* has various positive effects for the brand: it increases commitment to the brand (Tuškej et al., 2013), brand loyalty and brand advocacy (Stokburger-Sauer et al., 2012), and purchase intentions (Kuenzel and Vaux Halliday, 2008).

The consumer’s relation with a brand can manifest itself at different levels, dependent on the needs and goals of the consumer (Schmitt, 2012). At the object-centered level, the relationship is driven by a functional need to acquire information about and benefits from the brand. At the self-centered level, the relationship between brand and consumer is characterized by its personal relevance to the consumer. At the social level, the relationship is driven by the consumer’s desire of belonging to a community. While consumers can engage in relations with brands at one, two, or all three levels, the importance of the relation to the consumer increases from little meaningful at the object-centered level to intermediate meaningful at the self-centered level and, finally, to most meaningful at the social level. Brands can develop and foster these relations through advertising. Whereas traditional advertisements typically focus on product characteristics and service promotion, thus appealing to the object-centered level, brand stories are much more aimed at consumers and their individual and social identities, thus appealing to the self-centered and social level. The Pickwick story, for example, does not include explicit information about the tea product nor about the brand. Instead, it relates the individual experiences of a young girl in her efforts to improve the relationship with her family. The story thus communicates to consumers that the Pickwick brand enables them to be empathetic and brave individuals and, by implication, to create and maintain strong social bonds. As such, the story is targeted at both the self-centered and the social level of the consumer’s relation with the brand.

The concept of *archetype enactment* helps to understand the process via which brand stories affect the relation between consumer and brand (Woodside et al., 2008; Woodside, 2010). When enacting an archetype in a story, consumers adopt the role of a narrative character and vicariously experience in cognitive, emotional, moral or even embodied way, the narrated actions and events (Efthimiou, 2016; Sanders, 2017; Van Krieken et al., 2017). Enacting an archetype implies that the consumer is not a passive audience member, but, via simulation, an active participant in the story (cf. Mar and Oatley, 2008; Oatley, 2011). Because such participation is an intense form of story processing, it may result in positive outcomes for the consumer that stretch beyond the boundaries of the story into the daily life of the consumer, such as pleasure and sense making, which have been identified as the main outcomes of archetype enactment (Woodside, 2010). Although a single brand story can evoke either one or both outcomes, achieving “deep satisfying levels of sense making” is argued to be facilitated by pleasure, implying that sense making is of higher order (Woodside, 2010, p. 534).

Feelings of pleasure arise from the intense emotions experienced by the consumer and have therefore been described as *cathartic* in nature (Woodside, 2010; derived from the Greek noun *catharsis*). In the Pickwick story, emotional relief and happiness are felt when the young girl’s request visually stirs her mother and brings mother and daughter together. By contrast, sense-making is primarily rational and may arise from enacting the character’s examination of the self and others. Enacting the moral judgments narrative characters make with regard to their actions and decisions and those of others may cause consumers to evaluate and sharpen their own moral views. This outcome we describe as *phronetic* in nature: it is a form of moral sense making of the self that advances one’s practical wisdom and prudence [derived from the Greek noun *phronesis*; see Aristotle’s (2011) Nichomachean Ethics for an elaborate treatment of the concept]. The Pickwick story appeals to morality in two ways. First, the young girl implicitly characterizes her mother’s behavior as morally inappropriate in the sense that it harms the family bond. Second, in facing the difficult task of confronting her mother with her behavior in an attempt to reestablish the bond, she finds courage by accepting the practical help of a teabag label, by implication advocating the wisdom of drinking tea together; note that the brand’s slogan is Take the Time (Dutch *Neem de Tijd*). Consumers enacting the Hero archetype may be prompted to reevaluate their own family relations and, when considered necessary, may find themselves empowered to take action in a prudent way, for instance by taking the time to drink tea together and bringing up what is essential. Such an outcome is in line with the understanding that hero narratives fulfill two related functions: an *epistemic* function by offering scripts for prosocial action, by revealing fundamental truths about human existence and life paradoxes, and by cultivating emotional intelligence; and an *energizing* function by promoting moral elevation, inspiring psychological growth, and offering options and motivations to act (Allison and Goethals, 2016). In the Pickwick narrative, a script for confrontation and reconciliation is offered that presupposes a courageous and empathetic modus operandi; these traits in the story’s hero may be evoked and enforced in viewers when they imagine acting out a similar line of events in their own context. The quality of “becoming the Hero” via heroic enactment is thus strengthened if the use of archetypes from The Hero’s Journey resonates heroic traits and their corresponding moral values of the brand story’s narrative characters.

The Hero character traits and values in brand stories may be aligned with traits and values that have been associated with heroes and moral exemplars in the heroism literature (Allison and Goethals, 2016; Fagin-Jones, 2017). Allison and Goethals (2011) distinguish eight trait clusters of Heroes: smart, strong, caring, selfless, charismatic, resilient, reliable, and inspiring (the “Great Eight”; Allison and Goethals, 2011). From these traits, the Heroine character in the Pickwick narrative can be described as selfless (for being brave in revealing her deepest wishes while confronting her mother) and caring (for being empathetic and committed to the bond between her mother and herself), but also smart (for using a cleverly disguised strategy) and resilient (for enduring the previous period of detachment and staying committed).

The great diversity in brand stories, specifically in their expression of narrative archetypes, allows for different ways in which consumers’ archetype enactment can lead to cathartic and phronetic outcomes, and for resonating with various (combinations of) traits and values in heroic characters. Crucially, not all stages and characters of The Hero’s Journey have to be represented in a given narrative and, likewise, some stages and narrative roles may be emphasized more strongly than others. Understanding the relative importance of the various stages helps to understand the nature of archetype enactment pursued by brand stories. For example, a given brand story may invite consumers to enact the Hero while the brand is framed as the Helper supplying a Supernatural Aid, which is the case in the Pickwick story. Another brand story may invite consumers to enact the Helper archetype that helps the brand, represented as the Hero, to fulfill its journey. The brand as Supernatural Aid could be introduced during the resolution, supportive to the Hero in celebrating the completion of the quest, inviting the consumer to enact the reward of cathartic feelings such as *decreased vulnerability* and *satisfaction*. This type of enactment is evoked by the Pickwick story: during the story’s resolution, daughter and mother drink Pickwick tea together. Alternatively, or complementary, the Brand as Supernatural Aid can be introduced or foregrounded during the critical event, when it is supportive to the Hero in dealing with obstacles, inviting the consumer to enact phronetic experiences in which *wisdom* and *guidance* are central. This is also the case in the Pickwick story, in which the customized tea bag label is introduced during the story’s critical event: the young girl confronting her mother. In other words, the combination of a story’s archetypal structure and its narrative structure is meaningful and can be expected to influence the nature and outcome of the consumer’s archetype enactment.

Summarizing the previous, consumer enactment of brand story Heroes is likely to vary because of specific archetypal structures in the narrative plot and may be enforced by the narrative plot *showing* the story, as well as by the embedding of interactive voices *telling* the story. Depending on the interplay between showing the narrative plot and telling by narrative voices, enactment can invite consumers to develop feelings toward the brand that represent catharsis or phronesis, as well as moral values that align with heroic character traits that are reflected in the story plot. In the remainder of this study, we aim to explore how showing and telling interact in brand story videos to explain for various types of narrative enactment with brands’ archetypal Heroes. Following up on previous research entailing case studies (Papadatos, 2006; Ochoa and Lorimer, 2017; Laub et al., 2018), we present a conceptual framework that allows for an interdisciplinary and systematic investigation of brand stories. In this approach, the psychological models on archetypes and their representation are combined with psychological insights on heroic values and with narratological insights on perspective-taking and story structure. The resulting framework is applied in an initial analysis of 20 brand videos, aiming to assess its viability and to formulate expectations on possible consumer effects.

## Study

In order to gain more insight into the relation between brand stories and the nature and outcomes of archetype enactment, we conducted an analysis of a variety of brand stories. We showed the Pickwick brand story discussed in Section “Introduction” as well as a second, comparable brand story by Pickwick to a class of master students enrolled in a course on storytelling. We explained why these advertisements can be considered to be stories (rather than traditional non-narrative advertisements) and subsequently asked the students to select brand videos that they considered narrative and found to be attractive. This procedure allowed us to explore the extent to which brand stories that are *actually found* to be attractive by consumers display the archetypal structures that are *generally thought of* as being attractive. More specifically, it allowed us to determine the extent to which consumers are attracted to brand stories that show archetypal characteristics of The Hero’s Journey. This aim was informed by the widespread but yet to be tested contention that consumers, as well as audience members in general, are attracted to stories more than to other communication formats because stories are structured around archetypes that people can and want to relate to (e.g., Papadatos, 2006; Herskovitz and Crystal, 2010; Nelson, 2018). This procedure also enabled us to attain the second aim of our study: uncovering how the archetypal and narrative structure of brand stories act and interact in eliciting different types of archetype enactment, and exploring how this enactment may in turn allow for cathartic and/or phronetic outcomes.

### Collection of Brand Stories

A set of brand stories was collected by a group of 27 master students who were blind to the objectives of the study. Our aim was to assemble a representative set of at least 20 stories covering a reasonably broad range of brands. In small groups of two to four students, they were asked to collect videos that had been broadcast on television and/or posted online on corporate websites or social media channels. The students were instructed to select brand videos that met two criteria. First, they had to be *narratives* in terms of Labov and Waletzky’s (1967) criteria: the videos must entail a time lapse in which, at the minimum, time progresses from T_0_ to T_1_ and must include at least one character that could be identified as *subject of consciousness*, i.e., a character who consciously experiences the story events (Sanders et al., 2012). Second, the brand videos had to be *appealing* to the students.

### Analysis

First, the collected brand stories were categorized as either (1) singular plot stories or (2) embedded plot stories. A story was classified as a singular plot story if all story events took place on a single time line separate from the here and now. A story was classified as embedded plot story if it included interactive events taking place on multiple time lines that may, but do not necessarily have to be, connected to the here and now.

Next, the brand stories were analyzed on narrative structure (Labov and Waletzky, 1967; Labov, 2010) and archetypes (Campbell, 1949). To this end, the videos were first divided into fragments. The demarcation of fragments was primarily based on episode transitions, i.e., whenever the story’s setting changed in terms of time and/or place, this change was coded as the end of the current fragment and the beginning of a new fragment. Subsequently, each fragment was analyzed on a number of variables.

First, it was determined which element of Labov and Waletzky’s (1967) structure the fragment gave expression to. A fragment was coded as (1) the orientation if it was part of the introduction of the story’s characters and/or setting; as (2) a complicating action if it depicted one or more events central to the story plot; as (3) a critical event if it depicted the story’s peak, i.e., the crucial event that makes the story worth to be told; as (4) the resolution if it depicted what happened after the critical event, i.e., the outcome of the story; as (5) the coda if it depicted a transition from the story world to the here and now; and as (6) an evaluation if it expressed a verbal evaluation of or reflection on the story’s events. A single fragment could be coded as depicting multiple elements. Finally, if a fragment did not fit in either of these categories, it was coded as (7) none.

Second, the fragments were analyzed in terms of narrative archetypes and heroic values. The classification was a simplified version of Campbell’s (1949) the Hero’s journey. Taking into account the possibility that characters and objects can be presented as more than one archetype throughout the story, as well as the possibility that one archetypal role can be fulfilled by more than one character or object, the analysis was performed at the level of the fragment. For each fragment it was determined which narrative archetypes were depicted: (1) The Hero; (2) The Trickster; or (3) The Helper/Supernatural Aid. Based on Jung’s (1916) and Campbell’s (1949) descriptions of the various archetypes, a Hero was defined as the figure – either a person, object or concept – whose journey toward a specific goal is depicted. A Trickster was defined as a figure playfully deceiving or fooling the Hero. A figure was classified as a Helper/Supernatural Aid if it supported the Hero in overcoming obstacles and fulfilling the journey. A single fragment could depict more than one archetype. If the fragment did not display any archetypes, it was coded as (4) none. Each archetype was furthermore related to the actor performing the archetypal role, which could be (1) a character or (2) the brand. In addition, considering the story’s plot and the brand’s presumed goals with the story, it was determined which two of the eight basic heroic character traits and corresponding moral values – based on Allison and Goethals (2011) – were primarily reflected by the story’s Hero(es): smart, strong, caring, selfless, charismatic, resilient, reliable, and/or inspiring.

The aim of the above analytical steps was to assess the type of consumer enactment and value alignment pursued by the brand stories based on their narrative and archetypal structure. In the final step, the representation of the brand was analyzed. For each fragment it was determined whether the brand was represented by means of (1) a product; (2) a logo; (3) a slogan; or (4) not represented. A single fragment could be coded as including multiple brand representations. A schematic overview of the analytical procedure is provided in the **Appendix A1**.

### Procedure

The brand stories were analyzed by a team of three coders with experience in qualitative and quantitative narrative analysis. One coder first divided the stories into analytical segments and categorized them in terms of narrative elements (Labov and Waletzky, 1967). The segmentation and coding were subsequently discussed with the second and third coder; any disagreements were resolved during the discussion. In the next two steps, coder 2 and 3 worked independently to categorize each story in terms of narrative archetypes; character trait and moral value expression; and brand representation. Finally, both categorizations were compared and any differences were discussed and solved between coder 2 and 3, resulting in further refinements of the coding scheme.

## Results

### Brand Stories

The students’ search resulted in a total of 20 brand stories representing seven different brands: Heineken (2), Dove (2), Efteling (a fairy tale inspired amusement park in the Netherlands) (3), Innocent Drinks (2), Nike, Inc. (5), WNF (the Dutch branch of the World Wide Fund for Nature) (3), and Coca-Cola (3). Two movies (one by Efteling and one by WNF) had no narrative time line and were excluded from the analysis.^3^ The set was completed with two Pickwick stories^4^, resulting in a set of 20 stories covering 8 brands. In doing so, we aimed to arrive at a set that was on the one hand broad enough to represent a reasonable segment of narrative brand videos while it was on the other hand narrow enough to represent brand *stories* rather than other types of brand video advertisements. The length of the stories ranged from 30 s to 4 min and 24 s, with a mean duration of 1 min and 45 s. The videos had been broadcast on television or posted online between 2013 and 2018.

A total of 12 stories were classified as an embedding story, whereas the remaining 8 stories were classified as singular plot stories. **Table 1** shows an overview of both categories and provides for each story a short plot description as well as the brand’s goal.

**Table 1:** Plot descriptions of the singular plot and embedded plot stories.

Singular plots	Plot description
(1) Efteling: Time for Each Other https://www.youtube.com/watch?v=AEZDj6oiinc	A daughter of about 10 years old (=Heroine) in a family of four recreates the Efteling experience at home by decorating a room completely in the Efteling atmosphere, and in doing so she recreates the family’s experience of unitedness in pleasure and wonder such as they experienced at De Efteling.
	Brand’s presumed goal: evoking desire for togetherness connected to the Efteling experience, inviting the consumer to seek similar experiences with the brand.
	Reflected Heroic traits (moral values): caring and inspiring
(2) Efteling: The Efteling Amazes https://www.youtube.com/watch?v=kX1UBS27NNk	Max of about 8 years old (=Hero) introduces himself on his 1st day in a new class. He narrates about the unlikely adventures and achievements he made, receiving skeptical reactions of the teacher and classmates with one exception: a girl who understands that he is narrating his Efteling experience. Brand’s presumed goal: supporting, and evoking desire for, self-confidence and wonder connected to the Efteling experience, inviting the consumer to seek similar experiences with the brand.
	Reflected Heroic traits (moral values): charismatic and inspiring
(3) Nike: Choose go https://www.youtube.com/watch?v=6MgmbV5SbsA	A girl of about 20 years old (=Heroine) hears the news that the world has stopped turning. Inspired by her mouse who runs in his wheel, she starts running and encourages other people to join her in an effort to start the world’s turning again. This ultimately worldwide attempt succeeds, but runners appear to go the wrong way, and upon a new call they turn around to make the world turn the right way. At the end, the mouse in its wheel is shown again. Brand’s presumed goal: evoking enjoyment and promoting bonding by enabling the heroes’ enterprise.
	Reflected Heroic traits (moral values): smart and charismatic
(4) Coca-Cola: The Gardener https://www.youtube.com/watch?v=xgimZUJp5FM	A group of girls of about 20 years old tempts a young gardener (=Hero) to open a shaken can of Coca-Cola, thus wetting his t-shirt. He takes it off, wrings it out and goes on to mow the lawn, leaving the girls in awe. Brand’s presumed goal: evoke enjoyment and reflect sexual attractiveness in identification with (consumption of) the brand
	Reflected Heroic traits (moral values): charismatic and strong
(5) Coca-Cola: Thank your Christmas Hero https://www.youtube.com/watch?v=ScD_XiKlOLg	A boy of about 10 years old (=Hero) travels with a slay full of coca cola bottles and offers one secretly to people he encounters who are doing their best for others on Christmas eve, including – at the end – Santa Claus himself. Brand’s presumed goal: evoke enjoyment and promote altruism in identification with (consumption of) the brand
	Reflected Heroic traits (moral values): caring and inspiring
(6) Coca-Cola: Pool boy https://www.youtube.com/watch?v=iRigYg2dRZo	A brother and sister of about 20 years old (= Heroes) compete who reaches first with the pool boy to offer him a bottle of Coca-Cola. After overcoming various obstacles along the way, they finally face the pool boy, only to find out that their mother has already offered him a Coke. Brand’s presumed goal: evoke enjoyment and promote playful competition, sexual attractiveness and tolerance in identification with (consumption of) the brand
	Reflected Heroic traits (moral values): caring (empathy, tolerance) and inspiring
(7) Heineken: Worlds Aparthttps://www.youtube.com/watch?v=_yyDUOw-BlM&t=6s	Six adult characters of various ages and both genders (=Heroes) are, in antagonist couples, challenged to solve a build a construction puzzle that in the end appears to be a bar, at which they drink a Heineken beer together. The challenges they have to overcome involve sharing and understanding their mutual attitudes and private histories. Brand’s presumed goal: promote mutual understanding in identification with (consumption of) the brand
	Reflected Heroic traits (moral values): smart and caring (empathy, tolerance)
(8) Heineken: Champions League https://www.youtube.com/watch?v=-GBltOHBMj0	Three male characters between 25 and 30 years old, sitting on a terrace, are challenged to perform several soccer-related assignments striving for the missing part of a ticket to the Champions League Final. Their girlfriends, who are involved in the challenge, have the other half. In the end, the winning man (=Hero) is united with his girlfriend and the two have together one whole ticket. Brand’s presumed goal: evoke enjoyment and promote playful competition in identification with (consumption of) the brand.
	Reflected Heroic traits (moral values): strong and smart
**Embedded story plots**	**Plot description**
(1) Dove: Beauty on your own terms https://www.youtube.com/watch?v=_XOa7zVqxA4	Six women of various ages (=Heroines) narrate the challenges they had to overcome in accepting their own beauty on their own terms, despite critical comments. Brand’s presumed goal: supporting self-confidence by identification and representation of heroines.
	Reflected Heroic traits (moral values): charismatic and inspiring
(2) Dove: They said… https://www.youtube.com/watch?v=t7EjwAfMTPY	Jessica of about 30 years old (=Heroine) narrates how she, being a curvy beauty blogger, overcame the negative judgments of others on her physical appearance when she decided to start a beauty blog on her own terms.
	Brand’s presumed goal: supporting self-confidence by identification and representation of heroine.
	Reflected Heroic traits (moral values): selfless (courage) and inspiring

Embedded story plots	Plot description

(3) Pickwick: Beau’s Tea Topic https://www.youtube.com/watch?v=GBi77q-BY3s	Beau of about 12 years old (=Heroine) narrates how she and her mother have become estranged due to mom’s overusing her smartphone. She invites her mother to go for a picnic. When sitting down, she presents a tea bag with a personalized label asking her mother not to use her smartphone for 1 day. Mother and daughter discuss the matter over a cup of tea, agree and leave the scene arm in arm.
	Brand’s presumed goal: promoting confrontation and connection by enabling the hero’s enterprise and enabling the consumer to partake in similar missions
	Reflected Heroic traits (moral values): selfless (courage) and caring (empathy)
(4) Pickwick: Sanne’s Tea Topic https://www.youtube.com/watch?v=--kM4PXABnk	Sanne of about 25 years old (=Heroine) narrates how she used to bully Baukje, a former classmate. She invites Baukje to meet in their old classroom. When sitting down, she gives her teabag with a personalized label, asking to forgive her. Both women break into tears, embrace and discuss things over a cup of tea.
	Brand’s presumed goal: promoting confrontation and reconciliation by enabling the hero’s enterprise and enabling the consumer to partake in similar missions
	Reflected Heroic traits (moral values): selfless (courage) and caring (empathy)
(5) Innocent: Sustainable Bananas https://www.youtube.com/watch?v=grltlvtOJTo	Innocent (=Hero) narrates how it successfully sought for ways to deal with environmental and societal responsible banana growing companies.
	Brand’s presumed goal: promoting enduring sustainability by identifying their own enterprise and enabling the consumer to partake in this mission
	Reflected Heroic traits (moral values): resilient and reliable (social responsibility)
(6) Innocent: The Big Knit https://www.youtube.com/watch?v=T1b854BDX7w	Two elderly people, a woman and a man, narrate how they suffered from loneliness but have been brought together by Age UK, an organization supported by Innocent (=Hero) who contributes for each additional knit hat on Innocent bottles.
	Brand’s presumed goal: supporting altruism by enabling consumers to partake in this enterprise
	Reflected Heroic traits (moral values): caring (altruism) and reliable (social responsibility)
(7) Nike: Meet the runners: Lelisa Desisa https://www.youtube.com/watch?v=VOdYGKNMj7s	Lelisa’s coach narrates how Lelisa – a young and highly talented running athlete (=Hero) – is gradually growing toward being a prize-winning marathon runner.
	Brand’s presumed goal: supporting perseverance and self-manifestation by identification and representation of the hero
	Reflected Heroic traits (moral values): strong and resilient
(8) Nike: Unlimited Mo Farrah https://www.youtube.com/watch?v=_shug-k45Oo	Famous running athlete Mo Farrah (=Hero) narrates, supported by images of himself in his training facilities, what it takes for him – training far from his home for long periods of time – to be a prize-winning runner and why he makes the effort.
	Brand’s presumed goal: supporting perseverance and self-manifestation by identification and representation of hero
	Reflected Heroic traits (moral values): strong and resilient
(9) Nike: Unlimited courage: Chris Mosier https://www.youtube.com/watch?v=_gq8PO9XK2Y	Triathlon athlete Chris Mosier (=Hero) narrates, elicited by questions posed to him by a narrator during various training contexts, what uncertainties he – being a transgender – had to overcome when aiming for the men’s national athletic team.
	Brand’s presumed goal: supporting perseverance and tolerance by identification and representation of hero
	Reflected Heroic traits (moral values): resilient and caring (tolerance)
(10) Nike: Until we all win: Serena Williams https://www.youtube.com/watch?v=Ripg_LfJIeM	Multiple prize-winning tennis player Serena Williams (=Heroine) narrates, supported by images of successes and failures during her long career, what negative opinions of others she had to overcome to finally accept her own way of combining being a winner and being a woman.
	Brand’s presumed goal: supporting perseverance and self-confidence by identification and representation of the hero
	Reflected Heroic traits (moral values): resilient and inspiring
(11) WNF: Tiger Protector Pavel https://www.youtube.com/watch?v=2XOhBhKVtfU	Wildlife protector Pavel (=Hero) narrates how he makes an effort to protect tigers from poaching and maltreatment in the Russian wildlife, while he faces challenges such as severe cold and dangerous animals; in retrospective, he is shown to have lost a tiger due to poaching.
	Brand’s presumed goal: representing sustainability and altruism by identifying their own enterprise and enabling the consumer to partake in this mission
	Reflected Heroic traits (moral values): selfless (courage) and reliable (social responsibility)
(12) WNF: Tiger Protector Singye https://www.youtube.com/watch?v=V_u2h4oK_zQ	Wildlife protector Singye (=Heroine) narrates how she makes an effort to protect tigers from poaching in the Bhutan wildlife, while she is inspired by the Bhutan culture faces challenges such as dangerous poachers; in retrospective, she is shown to have been under the threat of poachers.
	Brand’s presumed goal: representing sustainability and altruism by identifying their own enterprise and enabling the consumer to partake in this mission.
	Reflected Heroic traits (moral values): selfless (courage) and reliable (social responsibility)


**Table 2** below shows the occurrence of the various narrative elements for the singular plots as well as the embedded story plots.

**Table 2:** Frequencies and percentages of singular plots as well as embedded story plots featuring the various narrative characteristics.

Narrative characteristics	Singular plots *N = 8*	Embedded plots *N = 12*	Total *N = 20*
Narrative structure
• Orientation	8 (100%)	12 (100%)	20 (100%)
• Complicating action	8 (100%)	11 (91,7%)	19 (95%)
• Critical event	8 (100%)	11 (91,7%)	19 (95%)
• Resolution	7 (87,5%)	9 (75%)	16 (80%)
• Evaluation	3 (37,5%)	12 (100%)	15 (75%)
• Coda	8 (100%)	12 (100%)	20 (100%)
Narrative archetypes^1^
• Hero	8 (100%)	12 (100%)	20 (100%)
Character	8 (100%)	10 (83,3%)	18 (90%)
Brand	0 (0%)	4 (33,3%)	4 (20%)
• Trickster	3 (100%)	0 (0%)	3 (15%)
Character	2 (66,7%)	0 (0%)	2 (10%)
Brand	2 (66,7%)	0 (0%)	2 (10%)
• Helper/Supernatural Aid	8 (100%)	12 (100%)	20 (100%)
Character	2 (25%)	4 (33,3%)	6 (30%)
Brand	8 (100%)	8 (66,7%)	16 (80%)
Heroic traits and moral values^2^
• Smart	4 (50%)	0 (0%)	4 (20%)
• Strong	2 (25%)	2 (16,7%)	4 (20%)
• Caring	3 (37,5%)	4 (33,3%)	7 (35%)
• Selfless	0 (0%)	5 (41,7%)	5 (25%)
• Charismatic	3 (37,5%)	1 (8,3%)	4 (20%)
• Resilient	0 (0%)	5 (41,7%)	5 (25%)
• Reliable	0 (0%)	4 (33,3%)	4 (20%)
• Inspiring	4 (50%)	3 (25%)	7 (35%)
Brand representation
• Product	8 (100%)	7 (58,3%)	15 (75%)
• Logo	8 (100%)	12 (100%)	20 (100%)
• Slogan	2 (25%)	6 (50%)	8 (40%)


*^*1*^The sum of the frequencies reported for brand and character may exceed the total number of stories because a given archetypal role may be fulfilled by both brand and character in a single story.*

*^*2*^For each story, the two most applicable heroic traits were distinguished.*

### Narrative Structure

**Table 2** shows that all brand stories in our set include an orientation and a coda, while not all show a resolution or evaluation. Typically, the singular plot stories had less explicit evaluations, because they show from the outside, rather than tell from the inside, what narrative characters experience. Noteworthy is that in all stories, a coda is part of the final fragment which signals a transition from the story world to the consumer’s reality, typically depicting the brand in terms of its product, logo, and/or slogan. In a minority of the stories (*n* = 3), the coda was the only element in the story that represented the brand. This was for example the case in the two WNF stories about tiger protectors. In these stories the main characters narrate their experiences in protecting tigers from poachers, describing more general, recurring complicating actions such as dealing with harsh nature conditions as well as specific events such as an encounter with poachers. While these specific embedded story plots include a resolution representing the outcome of the events, the overarching general stories lack a resolution. The absence of a resolution signals that the resolution lies beyond the boundaries of the story and has yet to take place in the consumer’s present or future. The coda shows the logo of the WNF, which functions as a bridge between the problems narrated in the story and the solution that the brand is searching for in the here and now. WNF invites the consumer to take part in this search by using the hashtag *#Iprotecttigers*. In other stories of our set, the brand is, in one form or the other, represented in all elements of the narrative. The stories by Nike serve as an example: in these stories, the brand is represented throughout in terms of its products that are worn by the characters. The brand thus visually supports the Heroes from the beginning to the end of their journey.

### Narrative Archetypes

The results show that two archetypes are present in all stories in our set: the Hero and the Helper/Supernatural Aid. In each of the singular plot stories, the role of Hero is fulfilled by a character. With the exception of one story, the Hero’s journey has a clear narrative structure including an orientation, one or more complicating actions, and a critical event followed by a resolution. Embedded story plots showed more variation than the singular plot stories, as the role of Hero in embedded stories can be fulfilled by the character, the brand, or both. In each of the Pickwick stories, for example, the role of Hero is fulfilled by the main character. By contrast, the stories by Innocent depict the brand as Hero. In the Innocent story about sustainable bananas, the brand represents itself as being on a journey toward building a fair company to be proud of. Uncertified banana farms fulfill the role of potential opponents, whereas certified banana farms function as Helpers for these farms protect the environment. In overcoming obstacles and establishing fruitful collaborations with certified banana farms, the brand is rewarded with the treasure of pride. Finally, in the two stories by WNF, a person working for the brand is depicted as the Hero. In these stories, both the character and the brand thus fulfill the role of Hero.

In the majority of the stories, the brand appeared to be represented as the Helper or Supernatural Aid that supports the Hero in facing difficult obstacles and completing the journey. For example, in one Coca-Cola story the goal of the main characters is to connect with the pool boy. Bottles of Coca-Cola help the characters in achieving this goal as they provide them with an excuse to approach the pool boy to offer him a dink.

Notably, only a small number of stories displayed the Trickster archetype and this archetype was only found in singular plot stories. In a different story by Coca-Cola, the Trickster role is fulfilled by a group of female characters who tricked the Hero – a gardener on a journey of mowing an enormous lawn – into being distracted from his task and opening a shaken can of Coca-Cola, thereby wetting himself. In this story the brand fulfills the role of Helper in two ways. First, the can helps the gardener to express his Heroism by providing him with an excuse to take off his shirt and and show his physical strength, thus impressing the group of women. Second, the can helps the women to connect with the gardener. Note that the Tricksters are, thus, Heroines as well, in the sense that they are on a journey toward being noticed by the gardener.

In the two singular plot stories by Heineken, the Trickster role is fulfilled by the brand. In both stories the brand deceives the Hero by setting up a game scenario of which not all crucial aspects are shared with the Hero, either in competition with Opponents for a Treasure (a Champions League Ticket) or facing challenges together (solving a puzzle). Notably, by playing the Trickster, the brand enables itself to take on the role of Helper/Supernatural Aid later on in the stories: after being tricked into a difficult quest, the Hero is invited to accept the help of the brand in completing the quest.

In sum, the results of our set reveal both similarities and differences between the various brand stories. All of its stories – both singular plot and embedded story plots – are structured around a Hero’s journey and feature a Helper or Supernatural Aid, a role typically fulfilled by the brand. A noteworthy difference that we found between the two types of stories is the limited use of evaluations in the singular plot stories and the frequent use of evaluations in the embedded story plots. These results could point toward an essential difference: in embedded stories, the *impact* of the Hero’s journey may be as important as the journey itself, as is made explicit in a combination of visual, auditive, and textual modes in the narration. This would imply that embedded story plots are structurally more complex, allowing the narrative elements and archetypes to be expressed at multiple levels.

### Narrative Enactment

Structural complexity in embedded brand story plots can be argued to evoke processes of enactment in consumers that differ from the enactment evoked by singular plot stories which are mainly visual by nature. For instance, in the Nike singular plot story “Choose Go,” few words are used to comment on many events, showing numerous characters involved in the attempt to make the world turn again. Although the story clearly features a central Hero – i.e., the girl who, inspired by her mouse’s wheel, initiates the running attempt – many other characters, anonymous and celebrities, are presented and available for identification. Most of the story’s actors do not say more than “Let’s go,” but they *show* that by going (in Nike sport gears), that the inconceivable is possible. Thus, they facilitate cathartic feeling of relief and amusement in viewers that can be enacted again when encountering the brand, hopefully to engage, as enthusiastically as did the Hero(es), in a movement of “going” that is facilitated by the brand’s running shoes, running garments, et cetera. The lack of evaluations maximizes the story’s potential to evoke catharsis: because the story plot is not interrupted by evaluative comments that are not part of the kernel story, consumers can become and stay immersed in the story world, co-experiencing the complicating actions, critical events and resolution as they unfold.

By contrast, Nike’s embedded story plot of Serena Williams “Until We All Win” merely implies Ms. Williams’ life story by showing numerous images of sportive episodes well known to the general public, while her voice *tells* how she overcame resistance and prejudice, to finally conclude that she has “proven time and time again, there is no right way to be a woman.” This message is heard while the visual shows Ms. Williams making a deep bow in acceptance of congratulations – suggesting surrender to her public and herself. Nike’s role here is (at least) twofold: to memorize it supported the actor in focus all through her career (thus, also in phases where she was challenged by resistance and prejudice); and by providing the actor a platform to present herself with her message. In viewing the images, consumers can acknowledge that they, too, may be “oversized” (and who *is* exactly the right size?) or “too mean if they don’t smile” (and who *does* smile all day, every day?); that they, too, may be “too black for tennis whites” (or too black, or brown, or white *for whatever outfit*) or “too motivated for motherhood” (or too occupied, or unfit *in whatever other sense*): in other words, that they, too, were thought (or thought themselves) to be a woman the wrong way. However, enacting victorious Serena, who was all that, they may prove for themselves and others that there is no one right way to be a woman; that there are in fact many right ways to be a woman, including one’s own, indeed: “until we all win.” This short but complex brand story shows a Hero’s life journey by imagery as well as tells the Hero’s moral message, causing phronetic effects of being brave and wise enough to surrender to one’s being, and thus of satisfaction and self-esteem. By facilitating such effects, Nike connects consumers to vital values on a deeper level than its sports garments: the Nike symbol is associated with diversity and self-confidence.

### Moral Value Alignment

In the brand stories, character traits are expressed by the Hero’s acts and intentions that reflect moral values with which consumers are invited to align their own values. For example, in The Big Knit, the brand’s presumed goal is to support altruistic and social responsibility values, classified as ‘caring’ and ‘reliable’ in terms of Allison and Goethals’ (2011) basic traits. Likewise, the Heineken Worlds Apart brand story promotes mutual understanding between characters, and in doing so represents empathy as the psychological and relational trait that is being mutually evoked to bond the other’ participants, and other people in general. By comparison, in the stories Pool Boy (Coca Cola) and Nike’s Chris Mosier, the brands implicitly promote the value of tolerance, regarding sexual orientation and transgender condition, respectively: again, the psychological and relational trait evoked here is empathy (classified as the trait *caring*).

All of the eight basic traits were found present in the analyzed set. The traits *strong, caring, charismatic*, and *inspiring* were found in both singular plots and embedded plot stories, while the trait *smart* was primary only in singular plot stories. For instance, the Nike Choose Go brand story depicts a Heroine who is both *smart* and *charismatic*: she thinks of a solution to get the world turning again, and succeeds in getting other people to run with her to achieve this. Likewise, the young men in the Heineken Champions League brand story need to be *smart* and *strong* in their competition for the match ticket. By contrast, the traits *selfless*, *resilient* and *reliable* were found only in embedded plots. For example, The Pickwick brand story of Sanne’s Tea Topic represents a courageous confrontation and reconciliation between characters; it combines the values empathy (trait: *caring*) with courage (trait: *selfless*). Likewise, the Innocent brand story of sustainable bananas combines the values of endurance and social responsibility (Fagin-Jones, 2017), categorized in terms of Allison and Goethals’ (2011) traits as *resilient* and *reliable*, respectively*;* also, the athletes promoted by Nike’s brand stories represent *resilience* in their enduring efforts to win, such as Serena Williams’ Until We All Win: “… but I have proven, time and again…” The occurrence of the values *selfless, resilient*, and *reliable* predominantly in the embedded plot stories is, to our view, not coincidental: in these values, the interests and needs of others are included. Such social value levels presuppose a higher degree of moral reflection on the self and others (Maslow, 1943). Examples are the value of social responsibility for sustainability and the courage to overcome fears for the self in the interest of others. In terms of story structure, the evaluative layer of an embedded plot allows for the explicit expression of such self/other oriented reflections.

### Scripted Narrative

An interesting finding is that some of the embedded story plots identified in this study represent a subgenre that suggests a journalistic style. These films typically include interview fragments that are interwoven with the story proper. Examples are the brand stories by Innocent, WNF, Dove, and Pickwick. These films continuously alternate between the Hero’s story events, shown as kernel story plot, and the interviews with the Hero which are conceptualized as evaluations that allow the film maker to “script” the Hero’s story plot. These alternations resemble the frequent shifts between news story and reconstructions found in journalistic reportages and newspaper narratives (Van Krieken et al., 2016). Temporarily moving out of the story proper into an explanatory mode serves in all these genres the same dual function: to lend the story authenticity and credibility and to engage the reader. This engaging function is fulfilled by the quotations of the characters giving expression to their evaluations and thoughts. For instance, in the Dove story of Jessica, images of the Heroine that show her leading a self-confident life are intertwined with interview fragments in which the Heroine tells directly to the camera – talking to an invisible, “journalistic” film maker – about the challenges she had to overcome in order to achieve the confidence of being beautiful on her own terms. Through the authentic Heroine, credible by her quotes and filmed in her own context, Dove facilitates a phronetic effect of mutual respect and satisfaction about beauty in various manifestations. A difference with news stories is that these “telling” interview scenes are in time positioned *before* rather than *after* the “shown” story scenes which are, in essence, planned and scripted but nevertheless trustworthy and authentic by means of legitimizing evaluations by the Heroine.

## Conclusion and Discussion

This exploratory study explains that in essence, brand stories are not about promoting products or services to customers but about establishing strong relations with consumers. Narrative archetypes have been argued to facilitate such bonding processes because they resonate with the audience and have the potential to establish mappings between the identity of the brand and the identity of the consumer (e.g., Ochoa and Lorimer, 2017). Our study provides support for the contention that consumers are indeed attracted to stories structured around archetypes (e.g., Herskovitz and Crystal, 2010), in particular the archetypal structure of The Hero’s Journey (Campbell, 1949). Moreover, the results of our analysis clarify how brands employ narrative archetypes and elements to foster the relation with consumers, both at the self-centered level of the consumer and the social level (cf. Schmitt, 2012), by enabling them to enact the roles played by the story characters. Brand stories were found to enable consumers to enact the journey of heroes toward pleasure or spiritual and moral enlightenment. Enacting these journeys and being vicariously part of the story world and its events may in turn influence the consumer’s life in terms of catharsis or phronesis, respectively. Whereas catharsis refers to deeply emotional experiences (Woodside, 2010), we have introduced the concept of phronesis to capture experiences of moral sense making that advance one’s prudence and practical wisdom.

**FIGURE 8:**
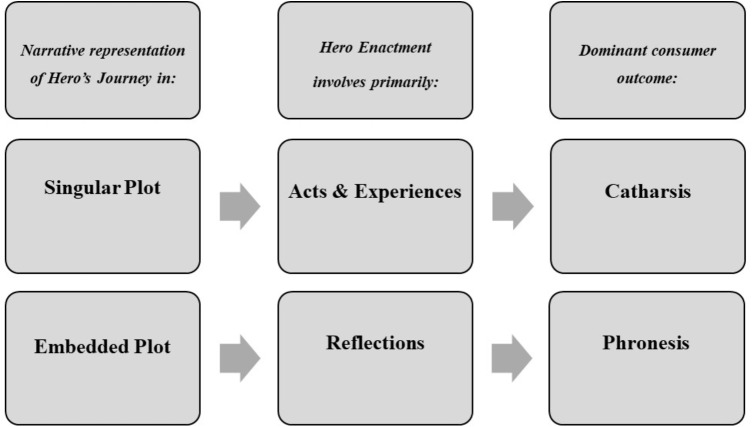
Hypothesized causal relations between narrative representation of Hero’s Journey, hero enactment, and dominant consumer outcomes.

Cathartic and phronetic outcomes of archetype enactment can be expected to strengthen the consumer-brand-relationship in distinct ways. Cathartic effects may be found for singular plot brand stories that show a narrative series of events, causing effects such as immersion into the story world and affective identification with characters, thus dominantly manifesting at the emotional level of the relationship between brand and consumer. Phronetic effects may be found for more complex brand stories that not only show, but also tell experiences, thus allowing consumers to simulate the Hero’s acts and experiences as well as the Hero’s inner reflections leading up to and resulting from these experiences. Thus, phronetic effects are likely to manifest at the emotional as well as rational level of the relationship between brand and consumer. Brand stories eliciting both catharsis and phronesis can be expected to have the strongest impact on the consumer-brand-relationship because they acknowledge that consumers, as individuals, are emotional as well as rational beings and such stories appeal to both sides simultaneously (see Grundey, 2008). Future research could test these expectations by assessing the causal relations between narrative structure, hero enactment and cathartic and phronetic outcomes as depicted in **Figure 8**.

Studies in this direction could combine online and offline measures to assess the nature of consumers’ hero enactment. For example, the use of fMRI techniques can provide insight into consumers’ enactment of the hero’s actions, galvanic skin response and heart rate measures can tap into the enactment of the hero’s emotional experiences, and implicit association tests can measure the enactment of the hero’s moral considerations (see Van Krieken et al., 2017).

Such studies could furthermore develop scales to measure cathartic and phronetic outcomes of the enactment processes. It is important to note, in this respect, that phronesis should be considered both distinct from and complementary to the concept of moral elevation. Moral elevation is strongly emotional in nature, a culmination of feelings of admiration and awe (Allison and Goethals, 2016, 2017). It results from the observation of heroic deeds – hence an outwards reflection – and may provide the observers with the inspiration and energy to adapt their behavior to the hero’s behavior in terms of moral considerations (Nakamura and Graham, 2017). By contrast, phronesis refers to an inwards reflection on one’s moral stance that results not from observing but from simulating heroic deeds. Alternatively, moral elevation could also be conceptualized as a complex process that in its ultimate form might result in phronesis.

Our present analysis indicates that some brand stories are intended, and in fact narratologically designed, to elicit phronesis rather than moral elevation. This can be explained by genre-specific goals: ultimately, consumers should be persuaded into buying the brand’s products or services after viewing the brand story. Stories about Heroes that are to be adored from a large distance are less suitable to achieve this goal compared to stories about enactable Heroes that, to specific target groups, may function as role models (Bandura, 2003); in other words, Heroes that the consumers can “be like,” “feel like,” and even “become” by thinking of, buying, or using the brand. The present study points toward two levels at which the brand enables the consumer to become a Hero. At the story level, the brand invites the consumer to enact heroic archetypes, supported by the supernatural aids that are provided by the brand. This enactment can be seen as a form of practice in preparing the consumer for journeys to be completed in real life (cf. Mar and Oatley, 2008; Boyd, 2009). At a meta-story level, the brand spends resources on the production of the story as a prerequisite for the consumer’s experience which at the same time may intensify this experience: consumers rely on the brand as the provider of catharsis and phronesis. Notably, the brand stories examined in the present study typically do not depict the brand as a Hero but as a supernatural aid that helps heroes achieve their goals. For consumers, enacting the journey of a Hero thus involves enacting an interaction with the brand; moreover, The Hero’s Journey cannot be completed without this interaction.

Some of the analyzed stories intervene at the social level, as they appeal to consumer’s imagination, or willingness to act in their own social context. For instance, the Pickwick stories invite consumers to customize their own tea bag label, while The Big Knit by Innocent expresses gratitude to customers who buy smoothies with an additional asset (a knitted hat) for additional costs, thus creating communities around the brand. Such transmedia enactment presumably establishes a stronger connection between the brand and consumers than forms of single medium enactment, as they invest more cognitive and actual resources in the interaction with the brand (Woodside, 2010; Granitz and Forman, 2015) and are inspired by the brand story to make particular choices that are not directly connected to the brand itself, but merely evoked by it through a combination of moral exemplars and invitations to act (compare Innocent’s The Big Knit). Note that Allison and Goethals (2016) found that of the Hero’s character traits and moral values, *inspiring* was rated highest by participants. Likely, such socially intervening brand stories allow consumers to build their identity by means of relations with brands at the most complex level, not only by buying, using and representing it, but also by ideologically internalizing it, which is reflected in the combination of indexical, iconic and ideological aspects of the consumer-brand relationship (Schembri et al., 2010).

This study’s contribution entails an interdisciplinary analytical model suitable for the systematic investigation of brand stories that combines narrative structure with archetypal characterization of brand story plots. As such, this study complements previous research on the archetypal structure of brand stories, as well as stories in general, which up until now has been dominated by case studies (e.g., Papadatos, 2006; Ochoa and Lorimer, 2017; Laub et al., 2018; for an exception see Delgado-Ballester and Fernández-Sabiote, 2016). Admittedly, an exploratory study as the current is limited in its generalizability of findings. Future studies conducting large-scaled analyses of brand stories should provide further tests of the framework’s viability and to arrive at a thorough understanding of how archetypal and narrative structures may evoke different forms of Hero enactment.

This limitation notwithstanding, the initial application of our framework to a varied set of complex narrative brand videos was helpful to clarify how brands and characters depict different archetypical roles to enact by consumers, that alternation between archetypical roles during the narrative is not uncommon, and that one role at different moments can be fulfilled by different actors. In addition, it clarified how brands have various narrative instruments available to target specific consumer groups with various effects. Brands may picture themselves as active narrative Helpers or Tricksters within story plots that invite consumers to enact Hero’s funny or enthralling adventures, causing cathartic effects of relief and joy in consumers, while other brands, or even the same brands, may alternatively play implicit, facilitating roles in multilayered narratives that invite consumers to enact Heroes’ liberating and satisfying emotions, causing phronetic effects that represent deeply shared moral values. Either way, the stories are intended to cause enactment that enforces brand-consumer connections, ultimately generating brand value.

## Author Contributions

Both authors listed have made a substantial, direct and intellectual contribution to the work, and approved it for publication.

## Conflict of Interest Statement

The authors declare that the research was conducted in the absence of any commercial or financial relationships that could be construed as a potential conflict of interest.

## Footnotes

1 https://www.youtube.com/watch?v=GBi77q-BY3s

2 JACOBS DOUWE EGBERTS, owner of Pickwick, has given permission to the authors to use figures and stills from the video “Pickwick: de Tea Topic van Beau” in this article. The authors hereby confirm that writing this article was their own initiative.

3 These brand videos depicted a great number of characters, each in one singular shot which at the most *implied* a narrative behind each pictured character, without actually narrating that story; these two movies were not analyzed.

4 The two Pickwick stories did not stand out from the other 18 brand movies: Fisher’s exact tests revealed no differences on the relevant categories *narrative structure*, *archetypes* or *brand representation* (*p*’s > 0.147).

## APPENDIX

**Table A1:** Appendix Analytical Coding Scheme.

Variable	Categories	
(1) Story type	• Singular plot story	
	• Embedded plot story	
(2) Narrative structure	• Orientation	
	• Complicating action	
	• Critical event	
	• Resolution	
	• Coda	
	• Evaluation	
	• None	
(3) Narrative archetypes	(3a)	(3b)
	• Hero	• Character
	• Trickster	• Brand
	• Helper/Supernatural Aid	
	• None	
(4) Heroic traits and moral values	• Smart	
	• Strong	
	• Caring	
	• Selfless	
	• Charismatic	
	• Resilient	
	• Reliable	
	• Inspiring	
(5) Brand representation	• Product
	• Logo	
	• Slogan	
	• None	
